# The Protection of Physicians during Epidemics

**Published:** 1882-01

**Authors:** 


THE PROTECTION OF PHYSICIANS DURING EPIDEMICS.—The occurrence of small-pox in an epidemic or quasi-epidemic form in several of our large cities at the present time, suggests the query whether some action cannot be taken to protect members of the profession in their relations to patients during the prevalence of contagious diseases.

While the means of protecting the public against the dire effects of small-pox still lack much to make them thoroughly effective, much has been and more is daily being done towards that end; but, so far as we are informed, no suggestions have been made looking to the protection of the conscientious physician in his relations to the public.

In the beginning of epidemics of small-pox, yellow-fever, cholera, typhus-fever, or similar contagious or infectious diseases, a morbid fear of being attacked prevails among all classes of the people. Should any physician be called to attend any cases of the disease at such times, he could not conscientiously refuse his services. If, however, the fact of such attendance becomes known to the physician's remaining patients, he is quite likely to receive hasty notices that his services in Mr. Smith's or Mr. Thompson's family can be dispensed with for the present. The doctor, of course, can read the meaning between the lines, and at once sees that he has been dismissed and has lost a good, and perhaps a highly valued patient by his generous, humane and conscientious imprudence. The poor small-pox or typhus-fever case, on the other hand, will probably die, and, instead of pay, the doctor gets a round share of abuse to console (?) him for the consciousness of having done his duty.

Can we blame the patient who has withdrawn his patronage, for failing to appreciate the unselfish devotion to duty of the physician? Clearly not. It matters little to him who else may be sick or dying of the contagious disease; he must see to it that the infection is kept out of his own household, if possible. Self-preservation is the first law to him, and we must acknowledge that he is justified in so regarding it. Leaving out of consideration the question of pecuniary loss to the physician, there remains the danger which the physician incurs of carrying the contagion into his own family, or the families of his other patients. This is not a "hypothetical question," and the danger is not exaggerated. It is a matter of frequent record, and is probably within the recollection of many of our readers that the contagion of small-pox, scarlet-fever and diphtheria, has been conveyed from house to house by the physician. A physician is not any more armed with an amulet or charm against the influences of contagion, than any other human being, and he should see to it that disease is not propagated through his fault or neglect. Some remedy should therefore be adopted which will avoid the dangers pointed out, while at the same time satisfying the scruples of the conscientious physician.

The following course, it seems to the writer, would be an appropriate one to adopt, and it is therefore urged upon the attention of the profession:

At the very beginning of an epidemic of contagious disease, however localized or insignificant, one or more competent physicians, who volunteer for the duty, should be appointed by the municipal or local health authorities to attend cases of the disease as they occur. The physicians so appointed, should be of such a character as to command the confidence of the community and their professional brethren. They should not allow any other business to interfere with the most thorough performance of their duties to the patients entrusted to their care. They should receive a stated salary, sufficient to enable them to decline general practice during the time they are engaged in their special duties as pest-doctors. The physician engaged in general practice should be discouraged from accepting cases of contagious disease, and be persuaded—it would not usually be difficult—to send all such cases to the official pest-doctor.

If some such measures as we have here outlined should be adopted, it would tend to limit the spread of contagious diseases, insure prompt and proper attendance upon those stricken down during the epidemic, protect the physician in his business relations with his patients, and finally secure an earlier report of, and more accurate registration of contagious diseases.

We believe the plan here sketched out is feasible, and that the municipal authorities of most of the cities now endangered, could be made to see its justice and propriety. Not only the members of the profession, but the general public likewise are deeply interested in any measures for the preservation of the public health. We believe that now is an appropriate time to agitate this measure, and hope some of our contemporaries will give it their attention.

